# Metabolomics-based study reveals the effect of lead (Pb) in the culture environment on *Whitmania pigra*

**DOI:** 10.1038/s41598-020-61745-1

**Published:** 2020-03-16

**Authors:** Xuemei Luo, Jieqin Meng, Xiufen Chen, Liangke Cheng, Shaopeng Yan, Luying Gao, Miao Xue, Yaojun Yang

**Affiliations:** School of Chinese Materia Medica, Beijing University of Chinese Medicine, Beijing, 102488 People’s Republic of China

**Keywords:** Biochemistry, Biogeochemistry, Environmental sciences

## Abstract

*Whitmania pigra*, called Mahuang (MH) in Chinese, has been used as a traditional Chinese medicine for many years and is susceptible to Pb exposure in aquaculture environments. To understand the impact of Pb in the culture environment on MHs, we carried out a 50-day culture of MHs in environments with different levels of Pb pollution. Then, tissue samples of MHs reared in the different Pb-polluted environments were collected and analysed by UPLC-Q/TOF-MS. The results showed that the Pb residue in MHs increased with increasing Pb in the culture environment. There was no significant difference in MH Pb content (*P* < 0.05) between the low-Pb residue group (PbL) and the blank control group (BC), and those of the middle-Pb residue group (PbM) and the high-Pb residue group (PbH) were significantly different from that of the BC group. Metabolomics results showed significant changes in 24 metabolites in the PbL, PbM and PbH groups, some of which were dose-dependent. These metabolites were mainly lipids, nucleotides, and dipeptides, which are involved in metabolic pathways such as glycerophospholipid metabolism, sphingolipid metabolism, and nucleotide metabolism. Overall, the results proved that metabolomics can be an effective tool to understand the effects of Pb on the metabolic responses of MHs.

## Introduction

*Whitmania pigra* Whitman (MH) is a medicinal leech that has been used as a traditional Chinese medicine (TCM) for more than 2000 years, and modern research has shown that its use has anticoagulation, antithrombosis, antiatherosclerosis and antiplatelet aggregation effects^1,2^. In recent years, the market demand for medicinal leeches has been constantly expanding, which has led to the rapid decline of wild medicinal leech populations as well as the constantly increasing price of medicinal leeches^3^. Therefore, in the aquaculture industry in Asia, farmed MH has become an important medicinal resource^4^.

*Hirudinea* species are benthic macroinvertebrates that live in shallow lakes, fishponds and pools^5^. Benthic macroinvertebrate communities are often used to assess the ecological quality of aquatic ecosystems^6^. Research has shown that MH is able to live in different saprobic levels in streams and trophic levels in lakes, but they usually prefer polluted environments^7^. Bioaccumulation of pollutants in *Hirudinea* was shown to be higher than in other benthic groups and higher than in fish in industrially polluted running waters^8^.

In an era of rapid industrial and economic development, heavy metals from human activities accumulate in the sediments of wetlands, affecting the organisms that live in or interact with them^9^. Zhao *et al*.^10^ investigated the spatial distribution (horizontal and vertical concentrations) of copper (Cu), lead (Pb), zinc (Zn), and cadmium (Cd) in five wetland types (a mudflat, an aquaculture wetland, a water area, a farmland wetland and a mangrove) from three areas (Ningde, Fuding, and Xiapu) of China. A study has shown that Pb is easily released from sediment into the water, and the morphology of Pb shows higher bioavailability compared to that of other studied metals (As, Cr, Hg and Ni), thus posing greater ecological risk^11^. China’s freshwater aquaculture model is dominated by pond culture^12^, and the cultivation of MH is mainly conducted in cultures; thus, the rearing of MH could be affected by Pb pollution in the sediments of the ponds. In addition, Pb is a non-essential element and has high toxicity, which can eventually accumulate in the human body through the trophic transfer of the food chain, affecting human health^13^.

Metabolomics is dedicated to the comprehensive analysis of the organic metabolites within a cell, tissue or biofluid that are naturally occurring and have low molecular weights^14^. The exposure of organisms to external stressors will induce changes in gene expression and protein production, both of which are affected by various homeostatic control and feedback mechanisms; moreover, these changes are magnified at the metabolome level^15^. This may make metabolomics a sensitive tool for evaluating the effects of external stress than other omics techniques. Environmental metabolomics is an application of metabolomics in characterizing the interaction between organisms and the environment, which allows for us to assess the actual physiological state of an organism^16^. Although nuclear magnetic resonance (NMR) spectroscopy is the primary bioanalytical tool for detecting metabolites in environmental metabolomics, mass spectrometry (MS) is beginning to revolutionize the detection ability in this field due to its higher sensitivity^17^.

In summary, as a widely used and economically valuable TCM, MH is susceptible to Pb exposure in the culture environment, which may affect its safety and effectiveness. However, no studies have been carried out to date on MH to evaluate the exposure and effects of Pb at the metabolite level. Considering these facts, this study selected UPLC-TOF-MS, which has relatively high sensitivity, to explore the metabolic perturbations in MH caused by Pb in the environment to identify biomarkers of exposure and toxicity mechanisms of action. Understanding the adverse effects of Pb on MH may provide more insights for preventing these effects.

## Results

### Pb residues in MH

The amount of residual Pb in the MHs of each group is shown in Table 1. The results show that there was no significant (*P* < 0.05) difference between the Pb concentration in the MHs of the low-Pb residue group (PbL) and that in the MHs of the blank control group (BC), and the Pb concentrations in the MHs of the middle-Pb residue group (PbM) and the high-Pb residue group (PbH) were significantly (*P* < 0.05) different from that in the MHs of the blank control group. The amount of Pb in the MHs increased with increasing Pb in the culture soil.

**Table 1:** Pb residues in MH under different culture environments (n = 4, $\bar{{\rm{x}}}$ ± s).

Group	Pb residual(mg·kg^−1^)
Blank control (BC)	2.05 ± 0.84
Pb low residue (PbL)	3.185 ± 1.63
Pb low residue (PbM)	6.46 ± 3.25*
Pb low residue (PbH)	10.66 ± 2.79*

^*^Indicates a significant difference compared to the BC group, *P* < 0.05.

### Results of metabolomics analysis of MH tissue

**Figure 1:**
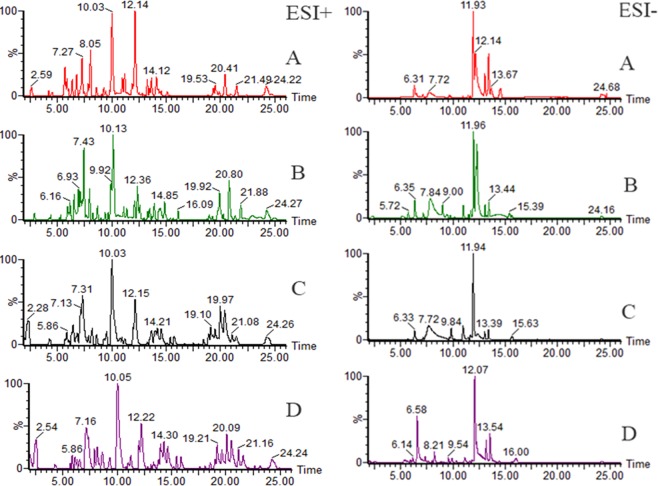
Base peak ion (BPI) chromatogram of tissue samples in each group in positive and negative ion mode. (**A**) BC group; (**B**) PbL group; (**C**) PbM group; (**D**) PbH group. These figures were created with Masslynx (version 4.1, https://www.waters.com/).

Base peak ion (BPI) chromatograms are shown in Fig. 1, and the BPI chromatograms for different groups show different peak shapes. In both ESI^+^/ESI^−^ ion modes, the peak profile of the BC chromatogram was similar to that of the PbL chromatogram, and that of the PbM chromatogram was similar to that of the PbH chromatogram. It can be preliminarily determined that Pb pollution will affect the metabolism of MHs. To illustrate the variation levels of metabolites in different groups clearly and accurately, it is necessary to further analyse the metabolomics data using pattern recognition methods.

**Figure 2:**
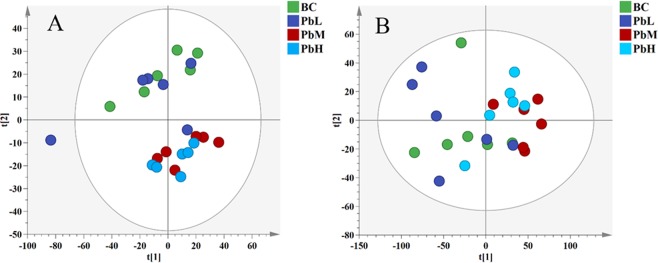
PCA score plots of MH tissue metabolites in positive (**A**) and negative (**B**) ion modes. These figures were created with SIMCA (version 14.1, http://www.umetrics.com/simca).

Principal component analysis (PCA) was first conducted to obtain an overview of variation among the groups (Fig. 2A,B). In ESI^+^ mode, the metabolite profile of the BC group was similar to that of the the PbL group, and the profiles of the PbM and PbH groups were similar. However, the distinction among groups regarding the metabolite profile was not obvious in ESI^−^ mode. Since PCA is an unsupervised method of analysis, not only the variables related to the group but also variables that are unrelated to the groups are included in the analysis. To filter those variables that were not related to the groups, we further analysed the variation among the groups with orthogonal partial least-squares discriminant analysis (OPLS-DA).

**Figure 3:**
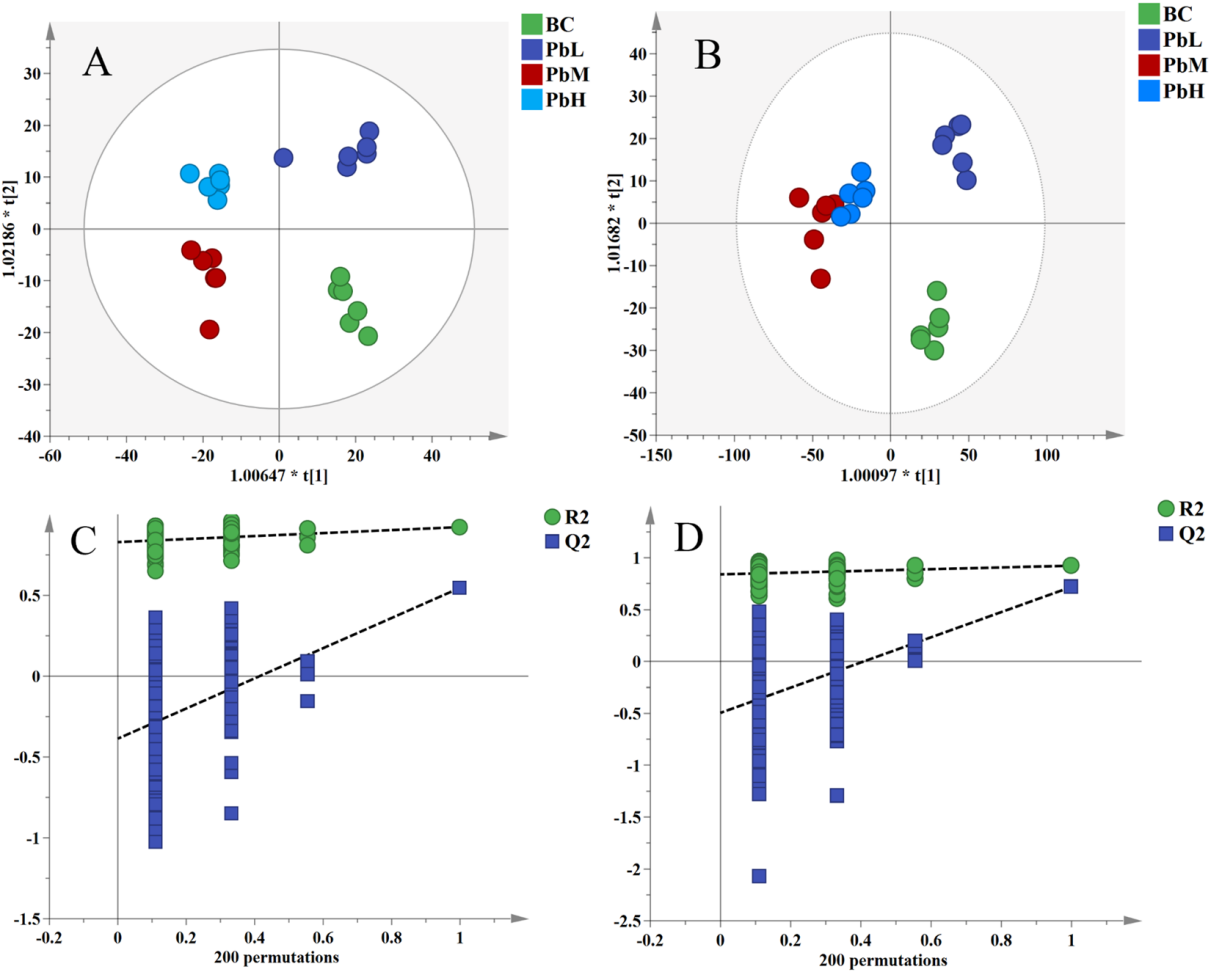
OPLS-DA score plots of MH tissue metabolites in positive ion (**A**) and negative ion (**B**) modes. The 200-time permutation test of the OPLS-DA model in positive ion (**C**) and negative ion (**D**) modes. These figures were created with SIMCA (version 14.1, http://www.umetrics.com/simca).

The OPLS-DA score plots (Fig. 3A,B) showed that the groups living in a Pb-polluted environment were separated from the control group in a dose-dependent manner, indicating that Pb in the aquaculture environment caused changes in the metabolites of MH. The model parameters were R^2^Xcum = 0.598, R^2^Ycum = 0.972, and Q^2^Ycum = 0.563 in ESI^+^ mode and R^2^Xcum = 0.79, R^2^Ycum = 0.979, and Q^2^Ycum = 0.539 in ESI^−^ mode, indicating good prediction ability and reliability of the model. Cross-validation of the OPLS-DA model was performed using 200 random permutations to evaluate the validity of the model. All Q^2^ values were lower than the R^2^ values, which indicated the excellent predictive capability of the model (Fig. 3C,D).

**Figure 4:**
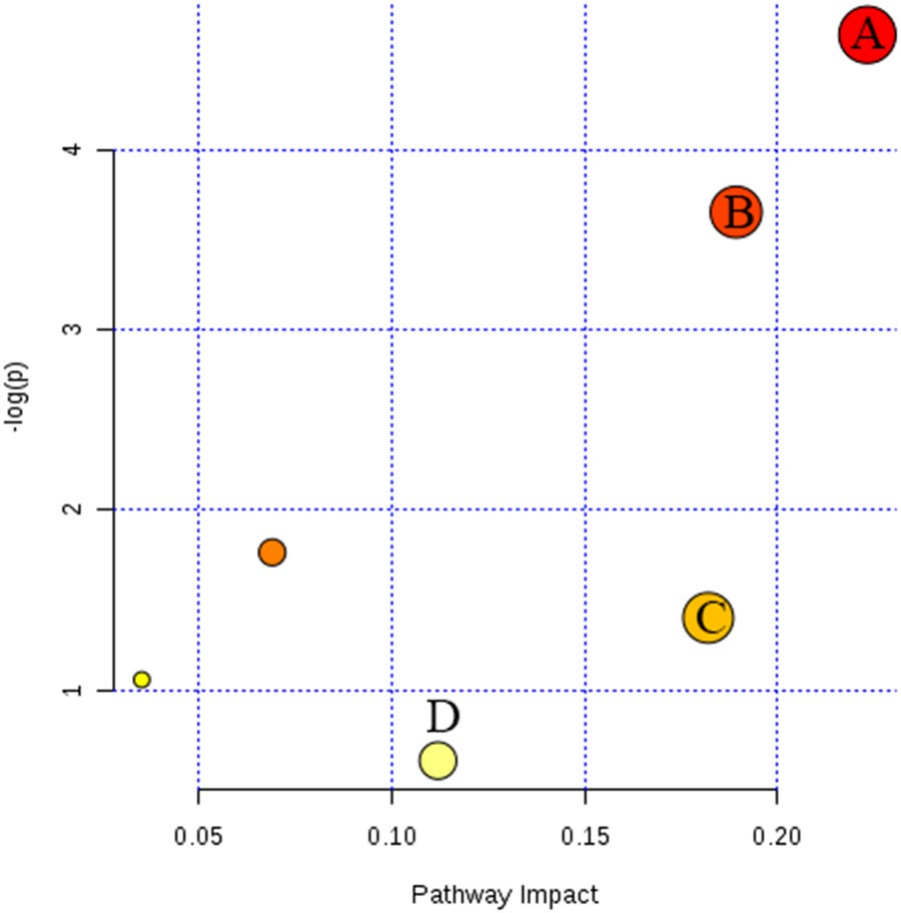
Metabolic pathway impact map of Pb on MHs. (**A**) Glycerophospholipid metabolism; (**B**) sphingolipid metabolism; (**C**) terpenoid backbone biosynthesis; (**D**) purine metabolism. This figure was created with MetaboAnalyst (version 4.0 http://www.metaboanalyst.ca/).

**Figure 5:**
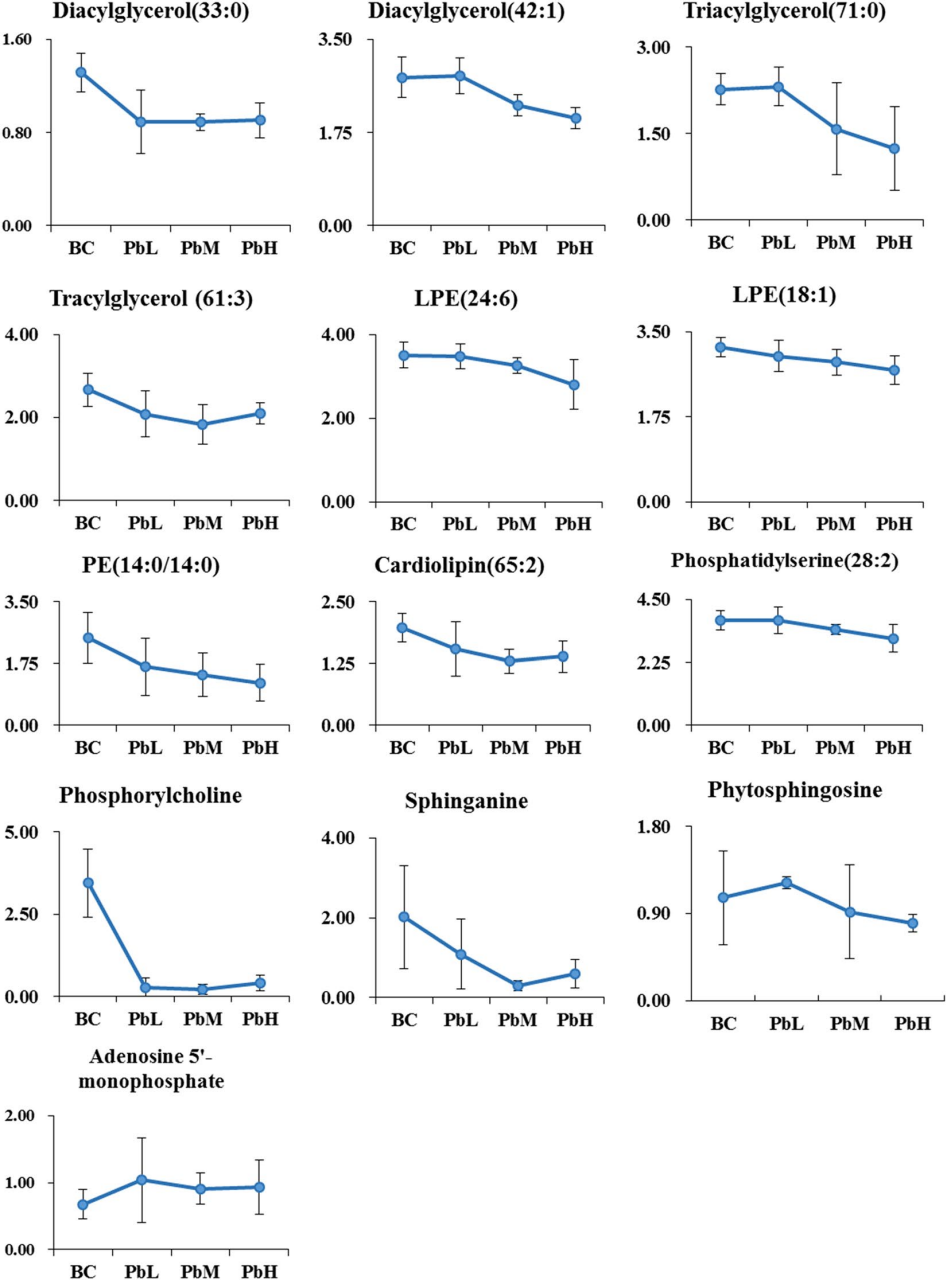
Changes in potential biomarkers in the BC, PbL, PbM and PbL groups.

The importance of each variance to the classification was determined by the value of variable importance in the projection (VIP). Metabolites with VIP values above 1.0 and *P*-values below 0.05 were considered differential metabolites. A total of 24 differential metabolites were found, and the results are shown in Table 2. To further elucidate the possible metabolic pathway affected by Pb, the above differential metabolites were introduced into MetaboAnalyst (http://www.metaboanalyst.ca/) to analyse the metabolic pathways. These differential metabolites were closely related to 6 metabolic pathways, as shown in Supplementary Table S2. It is considered that the pathway is important if the impact value is above 0.10 according to a ref. ^18^, so 4 metabolic pathways were obtained, namely, those of glycerophospholipid metabolism, sphingolipid metabolism, terpenoid backbone biosynthesis, and purine metabolism (Fig. 4). We selected dose-dependent differential metabolites as potential biomarkers (Fig. 5) from the above 4 pathways. The terpenoid backbone biosynthesis pathway was filtered out because the potential biomarker in it did not change in a dose-dependent manner. Finally, we found 12 metabolites of 3 metabolic pathways that were altered due to MH exposure to Pb.

**Table 2:** Characteristic information of differential metabolites of MH in Pb-contaminated environment.

NO.	Iron mode	classification	metabolites	Formula	RT/s	m/z	VIP score^a^	P-value^a^	Up or down^a^
1	ESI^+^	*Phospholipids*	PE(14:0/14:0)	C_33_H_66_NO_8_P	13.53	699.4705	3.26	0.0036	↓
2	ESI^+^		LPE(24:6)	C_29_H_48_NO_7_P	12.35	518.3062	1.10	0.0266	↓
3	ESI^+^		LPE(18:1)	C_23_H_46_NO_7_P	12.60	524.2758	1.73	0.0048	↓
4	ESI^+^		Phosphorylcholine	C_5_H_15_NO_4_P	0.97	184.0689	4.76	3.18E-07	↓
5	ESI^−^		Phosphatidylserine(28:2)	C_34_H_62_NO_10_P	9.63	656.393	1.88	0.03023	↓
6	ESI^+^		Sphinganine	C_18_H_39_NO_2_	10.89	274.2711	2.00	0.0110	↓
7	ESI^+^		Phytosphingosine	C_18_H_39_NO_3_	10.86	318.3058	2.02	0.0247	↓
8	ESI^+^		Cardiolipin(65:2)	C_74_H_140_O_17_P_2_	10.54	1376.9655	1.72	0.0024	↓
9	ESI^+^	*Triglycerides*	Tracylglycerol (61:3)	C_64_H_118_O_6_	10.44	996.8949	1.55	0.0208	↓
10	ESI^+^		Triacylglycerol(71:0)	C_74_H_144_O_6_	10.68	1143.1074	1.19	0.0393	↓
11	ESI^+^		Diacylglycerol(33:0)	C_36_H_70_O_5_	21.45	615.5587	1.66	0.0430	↓
12	ESI^−^		Diacylglycerol(42:1)	C_45_H_86_O_5_	10.43	1412.2859	1.38	0.0276	↓
13	ESI^−^	*Nucleotides and derivatives*	Uridine 2′-phosphate	C_9_H_13_N_2_O_9_P	1.07	323.031	1.26	0.0196	↑
14	ESI^+^		2′,3′-Cyclic UMP	C_9_H_11_N_2_O_8_P	1.04	348.0616	1.96	0.0013	↓
15	ESI^+^		Adenosine 5′-monophosphate	C_10_H_14_N_5_O_7_P	1.09	348.0613	2.00	0.0332	↓
16	ESI^+^		Cytidine monophosphate N-acetylneuraminic acid	C_12_H_16_N_2_O_3_S	11.36	1251.2936	2.00	0.0005	↑
17	ESI^−^	*Dipeptides*	Glutamylmethionine	C_10_H_18_N_2_O_5_S	7.71	243.0775	1.63	0.0476	↑
18	ESI^+^		Histidinyl-Isoleucine	C_12_H_20_N_4_O_3_	8.49	559.3003	2.68	0.0117	↑
19	ESI^+^		Phenylalanyl-Tryptophan	C_20_H_21_N_3_O_3_	7.96	393.1945	1.62	0.0281	↓
20	ESI^+^		Cysteinyl-Phenylalanine	C_12_H_16_N_2_O_3_S	1.05	301.1234	1.94	0.0001	↓
21	ESI^−^		Methionyl-Serine	C_8_H_16_N_2_O_4_S	1.08	273.0322	2.06	0.0332	↑
22	ESI^−^		Valyl-Arginine	C_11_H_23_N_5_O_3_	7.92	272.1746	1.32	0.0477	↓
23	ESI^−^	*others*	Farnesyl pyrophosphate	C_15_H_28_O_7_P_2_	9.13	427.1314	1.44	0.0040	↓
24	ESI^−^		N-Acetyl-D-Glucosamine 6-Phosphate	C_8_H_16_NO_9_P	1.04	300.0468	1.24	0.0062	↑

^a^The results of the group exhibiting the greatest change in the metabolite was compared with that in the BC group when the metabolite did not change in a dose-dependent manner, otherwise indicating the compared results of PbH with BC.

## Discussion

Statistical analysis of Pb residues in the MHs showed that there was no significant (*P* < 0.05) difference between the PbL and BC groups, and the PbM and PbH were significantly (*P* < 0.05) different from the BC group. However, the OPLS-DA results showed that the PbL group could also be distinguished from the BC group, indicating that measuring the Pb residue alone may not be accurate enough to determine whether the MHs are contaminated. The 2015 edition of the Chinese Pharmacopoeia stipulates that the Pb residue of MHs should not exceed 10 mg·kg^−1^, and MHs with Pb residues below this value are all qualified for use^19^. In this experiment, only the MHs in the PbH group exceeded the pharmacopoeia standards, but the metabolomic studies showed that Pb also affected the MH metabolism of the other groups, which may have an impact on the efficacy of the use of MHs. The metabolomics study also indicated that measuring the Pd residue in MHs alone may not be accurate enough to assess whether an MH is qualified for use or not. Metabolomics can be an effective complementary analysis for understanding the effects of Pb on MHs.

Metabolites were broadly grouped into the categories of *phospholipids, triglycerides, nucleotides, dipeptides* and *others*, which are mainly involved in energy metabolism, lipid metabolism, nucleic acid metabolism, protein breakdown, etc.

Heavy metals are well known to cause oxidative stress, which is characterized by the overproduction of reactive oxygen species (ROS), which impair the organism by attacking biological macromolecules and causing lipid peroxidation, DNA damage and protein oxidation^20^. Lipid peroxidation can cause mitochondrial damage and mitochondrial respiratory defects. In this study, we found that cardiolipin (CL) was significantly down-regulated in Pb-contaminated MHs. CL is a mitochondrial-specific phospholipid that promotes membrane fusion to support mitochondrial dynamics, maintain the overall mass of the mitochondrial population and increase mitochondrial respiration to maintain ATP production^21,22^. Therefore, the lack of CL can cause structural and functional abnormalities of mitochondria, such as affecting ATP production, and the organism may need to use alternative energy production pathways. Triglycerides are an important class of lipids that mainly exist as triacylglycerol (TAG) species whose main biological role is as energy storage depots^23^. TAGs are decomposed by enzymes into diacylglycerols (DAGs) and monoacylglycerols (MAGs) and, finally, into glycerol and fatty acids (FAs)^24^. FAs can be decomposed into water and carbon dioxide in the body and generate a large amount of energy, which is used by the body in the form of ATP^25^. In addition, studies have shown that fatty acid oxidation can also eliminate peroxidatively damaged lipids^26^. In this study, the TAGs and DAGs in Pb-contaminated MHs were significantly down-regulated compared to those in the MHs of the control group, indicating that Pb contamination caused oxidative stress in MHs, which affected their mitochondrial function and normal energy metabolism. Therefore, extra mobilization of fat was needed to maintain the normal physiological needs of MH. This result is consistent with previous research that showed that aquatic invertebrates need more metabolic energy to cope with stress in harsh environmental conditions, so their triglycerides will decrease^27^.

Glycerol phospholipids are the most abundant membrane lipid component in most eukaryotic cells, so homeostasis of the phospholipid class and acyl chains is critical for maintaining optimal physical properties of membrane that in turn are crucial for membrane function^28^. Membrane lipids are susceptible to oxidative damage, which changes the biophysical properties of cell membranes^29^. In the present study, phosphocholine (PC), phosphatidylserine (PS), phosphatidylethanolamine (PE), lysophosphatidylethanolamine (LPE), sphingosine and phytosphingosine were significantly down-regulated in the MHs exposed to Pb when compared to those in the MHs in the control group, which might indicate the use of these compounds to repair damaged membranes. The composition and integrity of the lipid membrane depend on the diversity of the phospholipid structure, where PE and CL facilitate the non-bilayer arrangement of the plasma membrane, and PC and sphingomyelin stabilize the bilayer structure^22^. These reduced phospholipids in this study may destabilize the membrane bilayer to nonbilayer arrangements, such as a hexagonal arrangement or inverted micelles, and these structures promote fusion and protein insertion in the membrane and are necessary for exocytosis^30^. Metal stress may also induce autophagy, which removes oxidatively damaged cellular components to resist oxidative stress^31^. Research has shown that the induced synthesis of PC and PE in the endoplasmic reticulum occurs simultaneously with the induction of autophagy and is the source of autophagosome membranes^32^. In our study, we found reduced PC, PE and overall glycerophospholipids, which may account for the organism using these phospholipids to form autophagosomes to remove stressors or damaged components and maintain homeostasis, thereby protecting the MHs exposed to Pb. Sphingolipids are not only used to construct membranes but are also involved in the formation of skin barriers, neural functions and apoptosis^33^. Sphingomyelin and N-acetylneuraminic acid are components of gangliosides, which have a significant promotion effect on nerve regeneration. Sphingomyelin and cytidine monophosphate N-acetylneuraminic acid were significantly down-regulated in this study, which may be the result of their use in the MHs to repair nerve damage caused by Pb. This is consistent with the results of a previous study that showed that chronic Pb exposure caused neurological damage to *Macrobrachium nipponense*^34^.

The altered profiles of uridine 2′-phosphate (UMP), 2′,3′-cyclic uridylic acid (2′,3′-cUMP), adenosine 5′-monophosphate (AMP) and cytidine monophosphate N-acetylneuraminic acid indicate stress-induced DNA/RNA damage. Nucleotides are precursors of DNA/RNA synthesis ^20^, so the up-regulation of UMP in this study indicated that Pb pollution caused gene damage in the MHs. Nucleotide degradation not only represents the negative effects of gene damage but also has a positive effect on metabolic homeostasis and resistance to oxidative stress^35^. AMP degradation helps maintain energy charges, and the purpose of AMP degradation is not to eliminate itself but to obtain carbon, nitrogen, phosphorus and other nucleotide-phosphoric acids, which are ultimately sent to the central metabolism to generate additional energy^35,36^. This explains the down-regulation of AMP and is also consistent with the conclusions obtained from the aforementioned changes in triglycerides. In addition, our research on metabolic pathways found that changes in nucleotides caused disturbances in the purine metabolic pathway. Studies have shown that abnormal purine metabolism has a destructive effect on the central nervous system^37,38^, which is consistent with the results caused by sphingolipid metabolism.

It is well known that metals can interfere with protein folding and are one of the main mechanisms of metal toxicity^39^. Stress conditions can cause protein misfolding, and if the proteins cannot be refolded, they will have to be degraded^39^. Changes in glutamylmethionine, histidinyl-isoleucine, phenylalanyl-tryptophan, cysteinyl-phenylalanine, methionyl-serine, and valyl-arginine may be the result of protein breakdown in the MHs caused by Pb exposure. Among these dipeptides, methionylserine has been reported as an antioxidant and supportive supplement^40^, and in this study, it may be used to combat oxidative stress caused by Pb pollution. In addition, a study^1^ has shown that the active ingredients in MHs may be macromolecular substances, such as proteins and peptides; therefore, protein breakdown may impact the efficacy of MHs as a traditional Chinese medicine, which should be further studied.

In summary, metabolomics has powerful potential as an effective tool for understanding the toxicological effects of environmental pollutants and determining their mechanisms of action. This study found that long-term exposure to Pb even at low concentrations can cause metabolic disorders in MHs, mainly lipids, nucleotides, dipeptides, and some other metabolites. Changes in these metabolites suggest that Pb contamination led to lipid peroxidation, gene damage, and protein breakdown in MHs, which may be due to oxidative stress caused by Pb. This self-protection mechanism in MHs promotes the repair of cell membranes (i.e., reduced levels of PC) and DNA/RNA (i.e., reduced AMP and UMP) and activates antioxidants (i.e., elevated methionylserine levels). Considering that nutrition manipulation can mitigate metal stress to some degree, the sufficient addition of nutrients, such as taurine, selenium and α-tocopherol^37^, may protect MHs against metal-induced stress.

## Methods

### MH sample acquisition

MHs were provided by the Breeding Base in Weishan Lake of Youbo Pharmaceutical Co., Ltd, Heilongjiang Province, China. The initial weight of the MHs was 10 g, and they were identified by Professor Yang Yaojun, the director of the identification department of Beijing University of Chinese Medicine. All of them were identified as *Whitmania pigra* Whitman. The maximum limit of Pb in the sediment (Pb ≤ 50 mg·kg^−1^) specified in the *Environmental Requirements for Origin of Non-Environmental Pollution of Aquatic Products* (GB 18407.4-2001) was used as the reference level^41^, and different concentrations of Pb(NO_3_)_2_ (batch number O5438, Beijing West Asia Chemical Co., Ltd.) was added to the pond soil in liquid form and mixed to implement soil Pb pollution modelling. The following doses were implemented: low-dose group, 50 mg·kg^−1^; middle-dose group, 250 mg·kg^−1^; and high-dose group, 500 mg·kg^−1^. The initial soil was used as a blank control. The content of Pb in the soil after modelling is shown in Supplementary material Table 1. The experimental soil was placed in an aquarium (60 × 30 × 35 cm^3^) with a soil thickness of approximately 2 cm. Fifty MHs were randomly placed in each aquarium and cultured with tap water. The culture temperature was controlled at 22–25 °C, which was monitored by an electronic thermostat in real time, and the oxygen was fixed and replaced for 2 hours every day^42^. The MHs were fed river snails (*Bellamya purificata*), and the feed residue was cleaned every day. The MHs were cultured for 50 days, some were dried at a low temperature for Pb residue detection, and some were stored at −80 °C for metabolomic studies.

### Detection of Pb residues in MHs

The dried MHs were powdered, passed through a No. 3 sieve, and 0.5 g of MH powder was accurately weighed. Then, 3 mL of HNO_3_ (Ruijing Chemical Co., Ltd, Suzhou, China) and 0.5 mL of H_2_O_2_ (Ruijing Chemical Co., Ltd, Suzhou, China) were added and digested in a microwave digestion apparatus (UltraWAVE, Milestone Company, Italy)^42^. The microwave power was set to 1300 W, and the samples were digested at 25 °C for 5 min, 150 °C for 5 min and 200 °C for 20 min. After digestion, the samples were cooled to room temperature and diluted to 15 mL with ultrapure water. Then, the samples were analysed by inductively coupled plasma mass spectrometry (ICP-MS) (DRC-II, Perkin Elmer GmbH, USA)^42^. The mass spectrometric operating conditions were as follows: nebulizer gas (Ar) flow rate, 1 L·min^−1^; plasma gas flow rate, 15 L·min^−1^; auxiliary gas flow rate, 1.8 L·min^−1^; and carrier gas flow rate, 1 L·min^−1^. The resolution was 0.7~0.9 amu, the dwell time was 100 ms, and single peak hop mode was implemented.

### Sample preparation for metabolomics

An MH tissue sample (50 mg) was precisely weighed, and then 1.5 mL methanol/water (1:1) that was pre-cooled overnight at −20 °C was added and homogenized in an ice bath. The homogenate was centrifuged at 12000 g for 10 min at 4 °C, and the supernatant was evaporated to dryness under a nitrogen stream (Lichen Bangxi Technology Co., Ltd., Shanghai, China). Then, the dry residue was redissolved in 120 μL of methanol/water (1:1), and particulates were removed by centrifugation. The supernatant was put into a vial and stored in a 4 °C refrigerator until analysis via UPLC-Q/TOF-MS^12^.

### UPLC-Q/TOF-MS conditions

The UPLC analysis was performed on a Waters ACQUITY UPLC System coupled to a Waters Xevo G2-S Q/TOF mass spectrometer (Waters Co., Milford, MA, USA). An ACQUITY UPLC BEH C18 column (2.1 mm × 100 mm, 1.7 μm, Waters Corporation, Wexford, Ireland) was used for chromatographic separation at 50 °C. The separation was performed with gradient elution using (A) water with 0.1% formic acid (v/v) and (B) methanol with 0.1% formic acid (v/v) as the mobile phase, and the flow rate was set at 0.4 mL·min^−1^. The following solvent gradient system was used: 99.9% A from 0.00–2.00 min, 99.9–75% A from 2–6 min, 75–20% A from 6–10 min, 20–10% A from 10–12 min, and 10–0.10% A from 12–21 min^12^. The autosampler was maintained at 4 °C. The injection volume was 5 μL.

Mass spectrometry was performed on a Xevo G2-S Q/TOF instrument with an electrospray ionization (ESI) source. The capillary voltage was 3.2 kV (ESI^+^) and 2.4 kV (ESI^−^), the sample cone voltage was 40 V for both ESI^+^ and ESI^−^ modes, and the extraction cone voltage was 6.0 V. The desolvation gas was set to 900.0 L·h^−1^ at a temperature of 350 °C, the cone gas was set to 25.0 L·h^−1^, and the source temperature was set to 120 °C. All data acquisition was controlled by Waters Masslynx (version 4.1) software (Waters, Manchester, UK).

### Data analysis

UPLC-Q/TOF-MS raw data were processed by XCMS online (https://xcmsonline.scripps.edu). After automated peak detection, retention time alignment and peak matching, the obtained data were normalized. Multivariate statistical analysis was performed by using SIMCA (version 14.1, Umetrics, Sweden). PCA is an unsupervised method of analysis that is usually used for variable reduction and separation into classes^43^. Partial least-squares discriminant analysis (PLS-DA) is a supervised extension of PCA that can identify the differential metabolites that account for the separation between groups^44^. Orthogonal signal correction (OSC) is a filtering method to decrease the within-class variability that is unrelated to class discrimination^20^; thus, in this study, the separation between Pb-exposed groups and the control group was maximized by implementing OSC-PLS-DA. To evaluate the validity of the established OSC-PLS-DA model, 200 permutation tests and repeated 2-fold cross-validation (2CV) were performed^45,46^. The quality of the model was assessed based on R2 and Q2 scores, as R2 explained the total variation of the data, and Q2 described the predictive ability of the model^47^. Differential metabolites were assessed based on VIP values (VIP > 1) and t-tests (P < 0.05)^48^. Differential metabolite information was entered into metabolite databases, HMDB (http://www.hmdb.ca) and KEGG (http://www.genome.jp/k:egg). Then based on the retention time and m/z values to identify the structures and chemical formulas of the metabolites to determine the potential biomarkers. Finally, the identified potential biomarkers were introduced into MetaboAnalyst (http://www.metaboanalyst.ca/) to analyse the metabolic pathways. Pb residues were analysed using SAS (version 9.4, One-way ANOVA), and multiple comparisons were performed using the LSD-t test.

## Supplementary information

Supplementary Information.
