# Supplementary material for: Metabolomics-based study reveals the effect of lead (Pb) in the culture environment on Whitmania pigra
Source: Sci Rep. 2020 Mar 16;10:4794. doi: 10.1038/s41598-020-61745-1 (PMC7075881; doi:10.1038/s41598-020-61745-1)
Supplement: Supplementary file 1 — Supplementary Information. [file 41598_2020_61745_MOESM1_ESM.pdf]

Metabolomics-based study reveals the effect of lead (Pb) in the culture environment on *Whitmania pigra*

Xuemei Luo, Jieqin Meng, Xiufen Chen, Liangke Cheng, Shaopeng Yan, Luyin Gao, Miao Xue, Yaojun Yang*

[1]School of Chinese Materia Medica, Beijing University of Chinese Medicine, Beijing, 102488, People's Republic of China.*Correspondence and requests for materials should be addressed to Y. Y. (email: yangyaoj002@sina.com)

Table S1. Lead content in cultured soils of each group (n=2)

	Lead content（mg·kg^{-1}）	
	Expected value	Actual detected value（ $\bar{x}$）
Blank control	0	7.59
Low dose	50	50.5
Medium dose	250	253.56
High dose	500	503.54

Table S2. Results of pathway analysis with MetPA

	Total	Expected	Hits	Raw p	Impact
Glycerophospholipid metabolism	31	0.47692	3	0.00963	0.2232
Sphingolipid metabolism	17	0.26154	2	0.025816	0.18919
Glycosylphosphatidylinositol(GPI)-anchor biosynthesis	12	0.18462	1	0.17098	0.06897
Terpenoid backbone biosynthesis	18	0.27692	1	0.24608	0.18196
Amino sugar and nucleotide sugar metabolism	27	0.41538	1	0.3472	0.03516
Purine metabolism	49	0.75385	1	0.54463	0.11193

Total is the total number of compounds in the pathway; the Hits is the actually matched number from the user uploaded data; the Raw p is the original p value calculated from the enrichment analysis; the Impact is the pathway impact value calculated from pathway topology analysis.
